# Feasibility of the Psychoeducational Programme SKILLS for the Child's Social Network for Patients Newly Diagnosed With ADHD: A Mixed‐Method Design Study

**DOI:** 10.1111/sjop.70034

**Published:** 2025-10-21

**Authors:** Martina Isaksson, Daniel Ekenberg, Elin Håkonsen Martinsen, Måns Lööf, Johan Isaksson

**Affiliations:** ^1^ Child and Adolescent Psychiatry Unit, Department of Medical Sciences Uppsala University Uppsala Sweden; ^2^ Division of Mental Health and Addiction, Child and Adolescent Psychiatric Department Vestre Viken Hospital Trust Drammen Norway; ^3^ Child and Adolescent Psychiatric Clinic Gävle Sweden; ^4^ Center of Neurodevelopmental Disorders (KIND), Centre for Psychiatry Research, Department of Women's and Children's Health, Karolinska Institutet and Stockholm Health Care Services, Region Stockholm Stockholm Sweden

**Keywords:** ADHD, adolescents, children, parents, psychoeducation

## Abstract

Attention‐deficit hyperactivity disorder (ADHD) presents challenges that both influence and are influenced by the child's environment. While non‐pharmacological interventions exist for youth and parents, brief and accessible programmes that also engage the wider social network are lacking. This study evaluated the feasibility of the psychoeducational programme SKILLS for the child's Social Network (SKILLS‐SN), focusing on implementation, acceptability, and preliminary outcomes. One hundred participants—including parents, grandparents, stepparents, and others in the child's network—attended the two‐session intervention at two sites, online or in person. Following the intervention, demographic data and satisfaction ratings were collected. Participants also completed pre‐ and post‐ratings of perceptions of the youth's ADHD, treatment, and challenges. Quantitative data were analyzed descriptively and with non‐parametric tests; an open‐ended response regarding opinions about SKILLS‐SN was analyzed with qualitative content analysis. Attendance was high, with over 97% completing both sessions; 17% of participants were non‐parents. Most rated SKILLS‐SN as good or excellent, and 99% would recommend it to others. Increased knowledge about ADHD was most valued. No significant changes were observed in participants' attitudes towards ADHD or treatment. The qualitative analysis identified three themes: programme strengths (e.g., useful as basic training), suggestions for improvement (e.g., more discussion time), and experienced impact (e.g., increased knowledge). SKILLS‐SN appears to be a feasible and acceptable brief psychoeducational programme for the child's social network. Future work should enhance participant interaction, broaden inclusion to school personnel and other key individuals, and further evaluate effectiveness and long‐term outcomes.

**Summary**

- SKILLS‐SN is a brief two‐session psychoeducational program for children's social networks after receiving an ADHD diagnosis.
- The program was feasible, with strong retention and successful implementation across two clinical sites.
- Acceptability was high: over 95% rated it good/excellent and 99% would recommend it to others.
- Participants valued increased ADHD knowledge, though no significant short‐term attitudinal changes were found.

## 1 Introduction

Attention‐deficit hyperactivity disorder (ADHD) is characterized by symptoms primarily involving attention deficits, impulsiveness, and hyperactivity (American Psychiatric Association 2022). The prevalence of ADHD has been reported to be approximately 3%–5% among children over 7 years of age and about 2%–4% among adults, with boys being three times more likely to receive the diagnosis than girls (Barican et al. 2022; Sacco et al. 2024; Salari et al. 2023; Simon et al. 2009; Thomas et al. 2015). However, other reports suggest an increase in ADHD prevalence during childhood (Chaulagain et al. 2023; Xu et al. 2018). In Sweden, the number of patients diagnosed with ADHD has risen significantly in recent years (Rydell et al. 2018), where recent data indicate that 10.5% of boys and 6% of girls between 10 and 17 years have an ADHD diagnosis (Socialstyrelsen 2023a, 2023b). A similar trend is seen in other countries, such as Denmark, where ADHD cases tripled among youth from 2006 to 2016 (Sundhedsstyrelsen 2017), and in Norway, where new figures from 2020 to 2022 show an increase in both genders, with the proportion of girls aged 16–24 years diagnosed doubling from 1.5% to 3.1% (Bang 2024).

Children and youth with ADHD often struggle with sorting, planning, remembering, and prioritizing, which complicates their ability to navigate a typical school environment and contributes to school‐related problems such as dropping out and academic underperformance (Barkley et al. 2006; Kent et al. 2011). Additionally, children and youths with ADHD often face interpersonal challenges, including difficulties with turn‐taking and communication (Jones and Hesse 2018; Wehmeier et al. 2010). They also frequently report lower quality of life compared to peers without ADHD (Klassen et al. 2004; Lee et al. 2016). Evidence indicates a significant impact on families and relatives of children with ADHD, both economically and in terms of psychological health (Flood et al. 2016; Fridman et al. 2017; Zhao et al. 2019). For example, parental depression is common when raising a child with ADHD (Cheung et al. 2018; Miranda et al. 2021; Zhao et al. 2019). Conversely, the child's surroundings—including family dynamics and school environment—can have both positive and negative effects on the course and severity of the child's ADHD symptoms (Claussen et al. 2024; Power et al. 2012; Shelleby and Ogg 2020; Wirth et al. 2019). This highlights a reciprocal relationship in which both the child's behavior and their environment continuously impact each other.

As the diagnosis becomes more prevalent, highlighting the challenges faced by a growing group of individuals, it is increasingly crucial to develop and identify effective and timely interventions that can be easily administered without long waiting times for this group and their support networks. Pharmacological treatment for ADHD primarily involves central stimulant medications such as methylphenidate, which are effective and considered first‐line treatments (Chan et al. 2016; National Institute for Health and Care Excellence (NICE) 2018). Although pharmacological treatments have been shown to be effective, they also have side effects (Felt et al. 2014; Greenhill et al. 2020), and a combination of drug treatment and psychosocial interventions may offer advantages for symptom reduction and functional outcomes (The MTA Cooperative Group 1999). Treatment guidelines recommend a multimodal approach that includes medications and psychosocial treatment (National Institute for Health and Care Excellence (NICE) 2018). Psychosocial treatments may include behavioral management interventions, cognitive interventions, skills training techniques, and psychoeducation, which can be provided both to children and adolescents with ADHD, as well as to those in their support networks (Evans et al. 2014).

Psychoeducation can be described as any intervention that educates patients and their families about their condition with a view to improving their long‐term outcome. Psychoeducation interventions can range from simply providing information about medication to enhance adherence (Peet and Harvey 1991) to more systematic, structured, and didactic information about the condition and its treatment (Rummel‐Kluge et al. 2006). The target of the intervention can also vary from educating the person with the condition to educating the entire family or only caregivers (Ogundele et al. 2023). Existing psychoeducational programmes for ADHD are mainly directed to parents or caregivers (Backman and Nytell 2014; Bai et al. 2015; Döpfner et al. 2004; Ferrin et al. 2014, 2020; Hantson et al. 2012; Montoya et al. 2011; Schoenfelder et al. 2020), leaving the extended network of the patient, such as teachers, peers, and extended family members, less addressed. While parental involvement is crucial, the broader social network—such as extended family, activity leaders, or other adults regularly interacting with the child—also significantly shapes the child's social and emotional development (Barreto‐Zarza et al. 2022). Inconsistent understanding or support across these individuals can undermine intervention efforts. Including several individuals that are important for the child in psychoeducation may improve cohesion in care, reinforce positive strategies in different contexts, reduce misunderstandings or stigma about ADHD, and, as such, build a social support network.

One example of a psychoeducational programme for parents of children and adolescents with ADHD is the Swedish programme STRATEGI. Research suggests that parental participation in such programmes may increase treatment compliance among youth with ADHD (Svanborg et al. 2009), and parents report increased knowledge and satisfaction after the programme (Backman and Nytell 2014). However, STRATEGI targets only parents and does not include the extended support network, such as teachers. Reviews of similar programmes (Dahl et al. 2020; Montoya et al. 2011) indicate that while many improve parental knowledge (Bai et al. 2015; Schoenfelder et al. 2020), effects on teacher knowledge are limited (Miranda et al. 2002), and no consistent impact on parental stress has been found (Ferrin et al. 2020; Svanborg et al. 2009). There are few programmes that have incorporated the extended social network of the child in psychoeducation. One exception is a programme described by Döpfner et al. (2004), which involved the child, family, and teacher—but this was delivered through individual sessions with a clinician, rather than in a group format. Another example is the Goal Management Training (GMT) programme, which has been adapted for adolescents with ADHD and incorporates a teacher component (Dyresen et al. 2024). Despite these efforts, psychoeducative programmes often fail to establish strong communication channels between parents, teachers, and mental health professionals, which are essential for consistent support.

In summary, there is some evidence supporting the effectiveness of psychoeducation programmes for youth and their families (Bai et al. 2015; Dahl et al. 2020; Ferrin et al. 2014; Lantz et al. 2021; Montoya et al. 2011). However, little is known about the impact of psychoeducational programmes available for other key individuals in the child's close environment who are not parents, as only a few studies have explored this area (Döpfner et al. 2004). Additionally, there is a lack of research on interventions that are both time‐efficient and accessible to many, including those living far from the clinic. This is of particular relevance for conditions such as ADHD, where psychoeducation often is regarded as the first line of treatment and hence should be offered for those newly diagnosed (Coghill et al. 2023). SKILLS for the child's Social Network (SKILLS‐SN) is one such programme, designed for relatives and other significant individuals in the child's life. SKILLS‐SN is derived from the psychoeducational programme SKILLS, originally designed for children and adolescents with ADHD (Lantz et al. 2021; Meyer et al. 2022). What makes SKILLS‐SN distinct is its explicit design for any significant adult in the child's network, regardless of formal caregiving role. This flexibility supports early access to psychoeducation in a group setting, an option for online participation, without requiring extensive clinician time, and helps build a common understanding across the child's everyday environments. As such, SKILLS‐SN is grounded in Bronfenbrenner's Ecological Systems Theory (Bronfenbrenner 1994), which underscores the importance of multiple environmental systems in a child's development, including connections between the child and others, as well as interactions among individuals within the child's settings. SKILLS‐SN is intentionally brief, consisting of two sessions, to ensure accessibility and minimize wait times following diagnosis. Most existing programmes are longer, with an average of six sessions (Dahl et al. 2020). This early intervention format aims to support families during the critical adjustment phase following diagnosis and foster shared understanding across everyday settings. The family can invite as many people as they want and repeat the programme as many times as they wish.

The aim of the present study was to evaluate the feasibility of the short psychoeducational intervention SKILLS‐SN, including assessments of implementation, acceptability, and preliminary outcomes. We hypothesized that: (1) SKILLS‐SN would be feasible to implement, as indicated by low attrition rates and consistent results across different geographical sites and age groups; (2) the intervention would be acceptable to the participants, meaning that they would find the programme appropriate and satisfactory; and (3) it would show some indications of preliminary outcomes in a positive direction, such as trends in more positive perceptions of the youth's ADHD and attitudes towards treatment after the intervention.

## 2 Materials and Methods

### 2.1 Participants

The study included relatives and other close individuals (*n* = 100) within the youth's network. The youth were up to 18 years old and had recently received a clinical diagnosis of ADHD. No exclusion criteria were applied, as the intervention aims to be accessible to as many individuals as possible. The participants were primarily parents, but also included grandparents, activity leaders, and step‐parents. See Table 1 for the demographic characteristics of the participants, including the diagnosis of the youth concerned. During the study period, 61 youths received an ADHD diagnosis at Site 1, and 115 at Site 2. At Site 1, a total of 106 relatives and significant others attended the SKILLS‐SN programme. Of these, 43 evaluation forms were submitted, corresponding to a participation rate of approximately 41% in the research study. At Site 2, 57 evaluation forms were collected, but the total number of programme attendees is unknown, making it difficult to determine the precise participation rate. Since several attendees sometimes filled out one form jointly, and families could invite multiple people, it is not possible to calculate the exact proportion of youths whose networks were represented, or the exact participation rate.

**TABLE 1:** Demographic characteristics of the participants.

	Site 1 (*n* = 43)	Site 2 (*n* = 57)	Total (*n* = 100)
Relation to the youth			
Mother	23 (54%)	31 (54%)	54 (54%)
Father	14 (33%)	18 (32%)	32 (32%)
Second‐degree relative	4 (9%)	8 (14%)	12 (12%)
Stepparent	1 (2%)	1 (2%)	2 (2%)
Foster parent	1 (2%)	2 (4%)	3 (3%)
Activity leader	1 (2%)	0 (0%)	1 (1%)
Gender of the youth			
Boy	27 (63%)	31 (54%)	58 (57%)
Girl	16 (37%)	25 (44%)	41 (41%)
Other	0 (0%)	1 (2%)	1 (1%)
Age of the youth			
0–12 years	26 (61%)	31 (54%)	57 (56%)
13–18 years	16 (37%)	26 (46%)	42 (43%)
Diagnosis			
ADHD, inattentive type	8 (19%)	10 (18%)	18 (18%)
ADHD, combined type	36 (84%)	47 (83%)	83 (82%)
Autism	11 (26%)	8 (14%)	19 (19%)
Other comorbidity (e.g., anxiety disorders, Oppositional Defiant Disorder)	7 (16%)	15 (26%)	22 (22%)

*Note:* Percentages may exceed 100% as respondents could select multiple options for the questions “Relation to the Youth” and “Diagnosis.” Percentages below 100% reflect instances of missing data.

### 2.2 Study Design and Procedure

The study employs a mixed‐method design that includes both a quantitative and a qualitative approach. This study is based on data from sessions conducted in two parts of Sweden: Region Gotland (Site 1) and Region Gävleborg (Site 2). The education was provided in child and adolescent psychiatric outpatient units and evaluated from autumn 2023 to late spring 2024. Families were invited to participate in the intervention following the psychiatric evaluation and diagnosis of their youth. They could sign up for the intervention either online or by phone. The goal was to have families participate in SKILLS‐SN as soon as possible after the ADHD diagnosis was confirmed. Participants were scheduled for the next available SKILLS‐SN session, typically within a few weeks of diagnosis. In some cases, families of children diagnosed earlier were also invited—either because the programme was not available at the time of their child's diagnosis or because they expressed interest in attending again. In both sites, it was possible to attend on‐site. At Site 2, where the distance to the clinic was significant for those living in other parts of the region, online attendance was also offered as a hybrid solution. In these cases, participants joined the session remotely from a local healthcare or municipal conference room, where the programme was streamed live. This setup was implemented to facilitate access while still providing a shared learning environment.

At the time of the intervention, all participants were informed about the study both verbally and in writing. There were no financial incentives or rewards to participate in the programme or the research study, and participation did not affect their current or future care. Those who consented to participate received evaluation forms to complete anonymously at the end of session 2. The evaluation form, which gathered demographic data, feedback on the programme, satisfaction with the programme, and an open‐ended question about their subjective perspective on the programme, was administered after the last session. Pre‐ and post‐measures, assessing participants' attitudes towards their youth's ADHD and potential treatments, as well as their perceptions of the problems associated with ADHD, were administered before the first session and after the final session.

### 2.3 The Intervention

SKILLS‐SN is a manual‐based educational programme designed by ML in collaboration with JI to be delivered shortly after an ADHD diagnosis. The purpose of the programme is to enhance knowledge about the diagnosis, offer strategies for managing everyday life, and provide information about available social support. The child's parents can invite anyone who has a relationship with the child. The programme is designed to accommodate both small and large groups, so that potential participants do not have to wait for a longer time. The material can also be used in individual sessions if needed. The education is delivered in Swedish, but materials are also available as pre‐recorded videos of the intervention with English and Arabic subtitles. An online format was available at Site 2, where the intervention was streamed live as a hybrid solution. The programme is conducted by a therapist in two 3‐h sessions, delivered 2 weeks in a row, incorporating PowerPoint presentations, discussions, and the exchange of experiences. The first session focuses on understanding what ADHD is, exploring treatment options (including medication, diet, sleep, and physical exercise), and providing information about available social and regional support. In the second session, the focus shifts to coping skills, covering everyday life skills as well as those relevant to family and school environments. The programme is recommended for the full spectrum of ADHD severity, from the mildest cases (where no medication is prescribed) to the most severe cases, where a more intensive array of interventions is advised. The lectures are recommended to be delivered by trained personnel with pedagogical skills who have undergone training in the SKILLS‐SN programme.

### 2.4 Assessment and Instruments

In line with recommendations for designing feasibility studies (Bowen et al. 2009), key areas such as implementation (i.e., attrition rates and whether different practitioners and locations would impact the outcome), acceptability (whether the intervention was perceived as appropriate and satisfactory), and limited‐efficacy testing (analyzing changes in participants' attitudes towards the diagnosis and potential treatment) were assessed.

#### 2.4.1 Evaluation Form

The evaluation form was specifically designed for this study. It comprised a quantitative section with 16 multiple‐choice questions, which included questions to confirm participation in both sessions, demographic information about the participant's relationship to the child, as well as details about the child's age, gender, and diagnosis/es. Additionally, four questions assessed whether participants had gained increased knowledge about their youth's ADHD, how to support youths with ADHD, available treatment options, and social support systems. One question explored what was gained from the programme within each of three areas – basic structure, rules and structure, and support and management, by asking: “What is the most valuable insight you gained from SKILLS‐SN regarding how to support your child based on their ADHD diagnosis?” The questionnaire also included questions on the participants' overall perception of SKILLS‐SN and whether they would recommend the programme to others. Responses were collected on categorical levels (e.g., Yes/No) and gradable ordinal scales (e.g., No, not at all/Just a little/Pretty much/Very much). At the end of the questionnaire, participants were given the opportunity to express their opinions about the programme in the form of freely written text. This open question was analyzed qualitatively and included in order to allow respondents to raise issues, thoughts, or experiences that we may have missed to ask. These responses may provide additional context which helps interpret the quantitative results more meaningfully.

#### 2.4.2 Pre‐ and Post‐Measures

Participants were asked to complete a questionnaire both before and after the programme. This questionnaire has previously been used in research (Lantz et al. 2021; Meyer et al. 2022) and included the following questions, which participants rated on a scale from 1 to 5 (Mostly negative/Somewhat negative/Neutral/Somewhat positive/Mostly positive), with a higher score indicating a mostly positive perception: *How do you perceive your child's ADHD? What is your attitude towards your child receiving pharmacological treatment for ADHD? What is your attitude towards your child receiving psychological treatment for ADHD?* Additionally, participants rated a final question on a scale from 0 to 10, with 0 indicating “Not at all” and 10 indicating “Very much”: *Does your child's ADHD lead to problems for him/her?*

### 2.5 Statistical Analyses

Statistical analyses were conducted and visualized using SPSS version 28 and R version 4.4.1. Answers regarding participants' experiences with the programme, including the knowledge gained and the benefits they perceived, were presented using bar charts, histograms, and descriptive tables. The Mann–Whitney *U* test and chi‐square tests were employed to assess differences in experiences between the two sites, between relatives of children of different ages, and between children with different diagnoses. Since the pre‐ and post‐measurements were not paired for each individual, the Wilcoxon signed‐rank test was not suitable. Instead, the Mann–Whitney *U* test was used to analyze differences before and after the intervention. Hedges' *g* was calculated as a measure of effect size, with values of 0.2 indicating a small effect, 0.5 indicating a medium effect, and 0.8 indicating a large effect size.

### 2.6 Qualitative Analysis

Qualitative analyses were conducted using content analysis with an inductive approach, as described by Graneheim and Lundman (2004). An inductive qualitative content analysis was chosen to explore participants' subjective experiences without imposing predefined categories. This method was deemed appropriate given the exploratory nature of the study and the open‐ended format of the data. First, two independent researchers (DE and MI) defined the aim of the analysis and familiarized themselves with the data from the first 54 participants, of whom 18 had provided written text. They then identified *meaning units*—defined as sentences or parts of sentences conveying how participants experienced the intervention. These were condensed and assigned descriptive codes. Codes with similar meanings were grouped into mutually exclusive categories. This process continued until the categories were clearly distinct from each other. Next, DE and MI compared and discussed their independently developed categorizations to refine the structure based on patterns and relationships in the data. They then incorporated the responses from the remaining 56 participants (17 with written text) to assess data saturation. These were independently coded and matched to the existing categories; no new categories were needed. The results were organized with clear descriptions of the categories and subcategories aligned with the study's aim and research questions. Lastly, the qualitative findings were compared to the quantitative findings and are further elaborated in the discussion.

## 3 Results

### 3.1 Demographic Characteristics

Relation to the youth and diagnosis were similar between the sites, confirmed by chi‐square analyses indicating no significant differences (*p* > 0.05). At both sites, it was more common to have a younger child (≤ 12 years) than an adolescent, being a mother was the most common relation to the child, and 15%–20% represented close others (not parents) to the child. Even though both sites had a higher representation of relatives to boys than girls, there was a statistically significant higher representation of girls at Site 2 than at Site 1, *X* ^2^ (1) = 4.88, *p* = 0.027. See Table 1 for detailed demographic characteristics of the participants.

### 3.2 Implementation

The SKILLS‐SN programme was implemented successfully across two clinical sites, with over 97% of participants attending both sessions at their respective sites, indicating strong retention. The intervention was offered both in‐person and as a hybrid solution, which facilitated participation from individuals across different regions. While exact coverage per diagnosed youth cannot be determined, attendance varied from one to several individuals per youth, suggesting good uptake within the broader social network.

### 3.3 Acceptability

#### 3.3.1 Analysis of the Experience of the Programme

**FIGURE 1:**
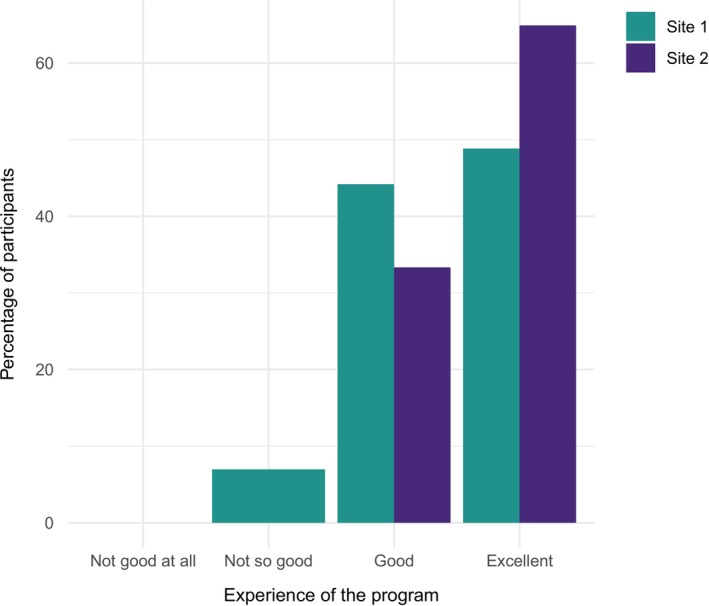
Presentation of the perceived overall experience of SKILLS‐SN, divided by site.

**FIGURE 2:**
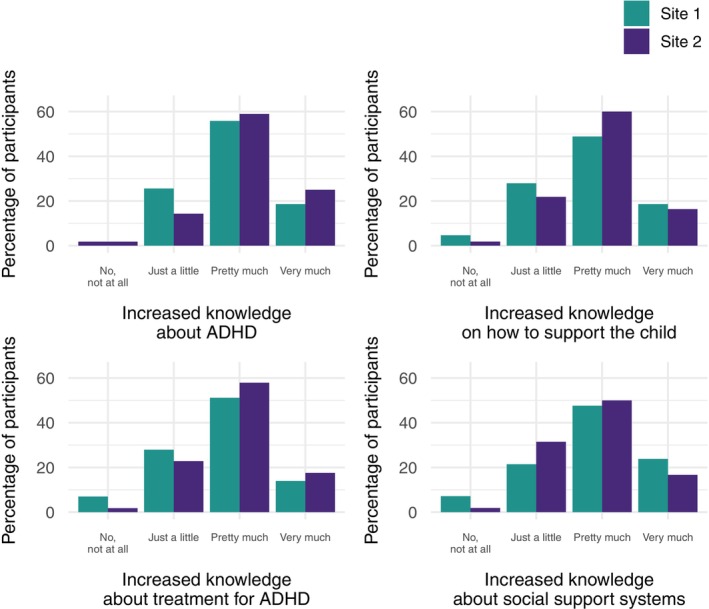
Perceived knowledge increases among participants after attending the SKILLS‐SN programme for relatives, divided by site.

All but one of those that participated in the evaluation would recommend SKILLS‐SN to someone else. Above 95% found the programme to be good or excellent at both sites (see Figure 1), and the majority found that they had increased their knowledge after the programme (Figure 2).

The Mann–Whitney *U* test compared the experience of the programme and the perceived increase of knowledge about ADHD, how to support their child, treatments for ADHD, and social support from society between the two sites, among different age groups (0–12 years and 13–18 years), and between parents and others in the youth's social network. No significant differences were observed; see Table 2.

**TABLE 2:** Comparisons of the overall experience of the programme and the perceived increase in knowledge after SKILLS‐SN using the Mann–Whitney *U* test.

Outcome	Site	*n*	Median	Mean rank	*U*	*p*
1. Experience of the programme	Site 1	43	3	44.5	968.0	0.053
	Site 2	56	4	54.2		
2. Increased knowledge about ADHD	Site 1	43	3	46.6	1059	0.250
	Site 2	56	3	52.6		
3. Increased knowledge on support	Site 1	43	3	47.8	1108.5	0.557
	Site 2	55	3	50.9		
4. Increased knowledge about treatment options	Site 1	43	3	47.0	1074.5	0.244
	Site 2	57	3	55.2		
5. Increased knowledge about social support systems	Site 1	26	3	50.4	1056.0	0.533
	Site 2	25	3	47.1		

Outcome	Age group	*n*	Median	Mean rank	*U*	*p*
1. Experience of the programme	0–12 years	56	4	58.6	1124.0	0.666
	13–18 years	42	4	50.7		
2. Increased knowledge about ADHD	0–12 years	56	3	46.2	990.0	0.132
	13–18 years	42	3	53.9		
3. Increased knowledge on support	0–12 years	55	3	50.1	964.5	0.123
	13–18 years	42	3	49.8		
4. Increased knowledge about treatment options	0–12 years	57	3	47.7	1189.5	0.953
	13–18 years	42	3	48.4		
5. Increased knowledge about social support systems	0–12 years	53	3	48.6	1097.0	0.897
	13–18 years	42	3	50.7		

*Note:* Answers were given on a scale from 1 (No, not at all) to 4 (Very much); ADHD (Attention‐deficit hyperactivity disorder).

#### 3.3.2 The Most Important Aspect Learned From the Intervention

**FIGURE 3:**
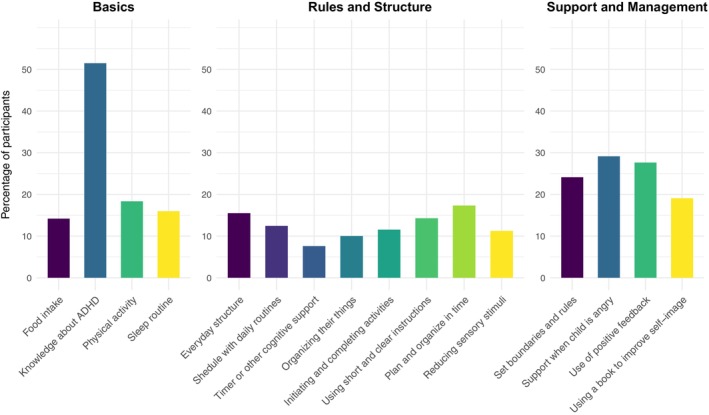
The most important aspects learned from SKILLS‐SN regarding how to support the child with ADHD across the categories of Basics, Rules and Structure, and Support and Management.

With regard to basic structures, most participants (87%) reported that increased knowledge about ADHD was the most important learning from the programme. For the other areas (rules and structure, and support and management), between 24% and 58% rated them as the most important (see Figure 3), with timer or other cognitive support being the least important, and supporting the child when angry and helping them plan and organize their time being the most important.

### 3.4 Pre‐ and Post‐Intervention Measurements With Limited Efficacy Testing

Pre‐ and post‐ measurements of attitudes towards ADHD, its challenges, and its treatment were analyzed. A Mann–Whitney *U* test showed that no significant differences were observed, and effect size calculations showed at most small effects; see Table 3. Analyses of pre‐ and post‐ measures where the sites were looked at separately were also performed. The results were essentially the same, i.e., no significant differences were observed.

**TABLE 3:** Summary statistics and comparisons of perceptions and attitudes towards ADHD, related problems, and ADHD treatment before and after the intervention.

Question (min value‐max value)	Time	Mean	Median	*n*	Mean rank	*U*	*p*	Hedges'*g*
1. How do you perceive your child's ADHD? (1–5)	Pre	2.14 (1.00)	2	88	68.94	2150.5	0.418	0.23
	Post	2.40 (1.31)	2	53	74.42			
2. What is your attitude towards your child receiving pharmacological treatment for ADHD? (1–5)	Pre	3.84 (1.29)	4	88	67.77	2057.5	0.195	0.25
	Post	4.15 (1.12)	5	53	76.37			
3. What is your attitude towards your child receiving psychological treatment for ADHD? (1–5)	Pre	4.64 (0.75)	5	85	71.40	1921.0	0.156	0.17
	Post	4.51 (0.76)	5	51	63.67			
4. Does your child's ADHD lead to problem for him/her? (1–10)	Pre	7.18 (2.08)	8	88	72.45	2116.0	0.449	0.06
	Post	7.06 (1.89)	7.5	52	67.19			

*Note:* Answers for items 1–3 were given on a scale from 1 (mostly negative) to 5 (mostly positive). Answers for item 4 were given on a scale from 1 (not at all) to 10 (a lot). ADHD (Attention‐deficit hyperactivity disorder).

### 3.5 Qualitative Analysis

In total, 35 of the 100 participants expressed their opinions about the programme in the form of freely written text. The qualitative analysis of this text resulted in three themes and nine subthemes. A detailed table of the themes, subthemes, and participants' comments can be seen in Appendix S1.

#### 3.5.1 Strengths of the Programme

The participants expressed various kinds of positive feedback, several of them coded into a subcategory of *Appreciation for the programme and its educators*, which included reflections that did not pertain to any particular part of the education such as: “Very good”, “Satisfied as a whole, a good programme!” and “Fantastic leaders”. In the subcategory *Good structure and content*, experiences such as “Good structure, clear, good mix of information/facts/community support/book tips. Thank you”, and “Very good length of the programme” were expressed. In the subcategory *Good as introduction and repetition*, participants claimed the programme was easy to understand and was a good introduction to learn about ADHD: “Good as a foundational/basic training if you don't have much knowledge”.

#### 3.5.2 Suggestions for Development and Improvement

Some comments were grouped under a theme for suggesting improvements. A part of these pertained to the subtheme *Physical environment*, including remarks about the technical solutions such as “Better/larger screen needed for the speaker/instructor, better sound. Could have been connected from home. Better with two large screens”. In the subcategory *Room för discussion*, participants expressed a need to also be able to interact with the other participants: “…I believe there is a need to discuss and learn from each other. It can be a bit challenging to just sit and listen. Maybe more opportunities should be provided for talking/discussion”. In the subtheme *Extending the content*, participants expressed a variety of suggestions for improving the content, such as “From an activity‐leader perspective I wish for more info from other perspectives than just parents” and “My child got their diagnosis very late, feels like the education is directed towards children that have been ‘noticed’ earlier”. In the subcategory *Distribution to schools*, several suggested that SKILLS‐SN should be distributed to school personnel: “Wish teachers would take part in SKILLS”.

#### 3.5.3 Impact of the Programme

Even though the task was to give opinions about SKILLS‐SN, some participants left comments regarding what they learned from the programme. Under the subtheme *Social and psychological benefits*, participants expressed how the programme affected their wellbeing with comments such as “The knowledge I have received during SKILLS has decreased my stress!” and “I have gained knowledge that has resulted in a better relationship with my daughter (who has ADHD)”. In the subtheme *Increased knowledge*, participants highlighted similar experiences as in the quantitative data, such as “Good for everyone to attend in order to increase knowledge about ADHD and how to approach the child”.

## 4 Discussion

The aim of the present study was to explore the feasibility of the psychoeducational intervention SKILLS‐SN, including assessments of implementation, acceptability, and preliminary outcomes. Our main findings indicate that SKILLS‐SN appears to be a feasible intervention programme, as it was successfully implemented in two clinical outpatient settings and was deemed acceptable among participants, supported by both qualitative and quantitative data. Another important finding was that almost all participants would recommend SKILLS‐SN to others. However, the programme did not demonstrate preliminary effectiveness regarding participants' attitudes towards their child's ADHD or its treatment, and some participants reported the need for more sessions to allow time for discussion and highlighted the importance of also including school staff and others beyond the immediate family.

Regarding implementation and adaptation to different sites and age groups, the results of the present study indicate that the intervention is feasible. This conclusion is supported by high retention rates from session one to session two and the fact that many of the diagnosed participants had several individuals within their social network that participated in the programme. The programme's flexibility—being offered both from distance and on‐site, with short waiting times, in contrast to many other psychoeducational programmes for ADHD (Dahl et al. 2020; Montoya et al. 2011)—likely contributed to its successful uptake across two diverse clinical settings. As noted by Bowen et al. (2009), successful implementation is itself a core dimension of feasibility, encompassing factors such as demand, acceptability, and practicality. In this study, SKILLS‐SN was well received at both sites, retained nearly all participants, and was adaptable to different delivery formats and participant roles. These findings therefore support the feasibility of implementation and offer early insight into potential scalability. However, only one‐fifth of participants represented non‐parental roles, highlighting challenges in reaching broader network members. This has implications for future adaptation of the programme's outreach strategies. The qualitative data analysis indicates a desire for greater involvement of teachers, which aligns with research suggesting that interventions should be directed towards those surrounding the child (Aguiar et al. 2014; Ferrin et al. 2020; Kirby 2015; Ward et al. 2022). Allowing for individual online participation might attract participants with less close relationships to the child and could also increase the average number of participants related to each child. As stated in the Ecological Systems Theory (Bronfenbrenner 1994), a child's development is influenced by multiple layers of their environment, supporting the idea that psychoeducation should extend beyond parents to the wider social network. Another aspect of successful implementation is that the intervention has been shown to be equally effective across different sites and age groups, and it is adapted to various settings (Bowen et al. 2009). Our analyses showed no statistical differences between the two sites in terms of overall satisfaction. In terms of gender, a higher proportion of the participants were relatives of boys than girls, which is reasonable given that more boys receive the diagnosis of ADHD (Polanczyk et al. 2007; Salari et al. 2023).

Regarding acceptability, all but one participant would recommend the programme to others, and over 95% rated SKILLS‐SN as good or excellent. This was supported by comments analyzed using content analysis, stating a general satisfaction with the intervention, which was regarded as easy to understand and inspiring. Furthermore, a vast majority of the participants felt they had gained increased knowledge about ADHD, which was also regarded as the most important aspect by a majority. The findings align with the principle of the health belief model, which posits that the likelihood of using health behaviors is shaped by an individual's awareness and comprehension of their condition (Champion and Skinner 2008). However, some participants expressed a desire for deeper knowledge and additional sessions. Participants also rated increased knowledge about how to support their child, available treatments, and social support systems after completing the programme. These findings correspond to previous research in the field (Lantz et al. 2021; Lopez et al. 2005; Schoenfelder et al. 2020). Information about everyday structuring, planning and organizing time, providing short and clear instructions and positive feedback, and setting boundaries and rules were all rated as important subjects in SKILLS. These subjects are also included as central principles in parental guidance programmes for children and adolescents with ADHD and conduct problems (Hagen et al. 2011; Patterson et al. 2002; Sinus AB n.d.; C. Webster‐Stratton et al. 2013; C. H. Webster‐Stratton et al. 2011).

The qualitative analysis also revealed themes reflecting on the acceptability of the programme, i.e., what participants appreciated and what needed further development. Strengths of the programme included its usefulness as an introduction to learn about ADHD, the benefits of repetition, the skills of the instructors, and the clarity of the educational content. In the theme “Suggestions for development and improvement” participants offered both constructive feedback and positive suggestions for expanding the programme's reach and content. Key suggestions included extending invitations to the intervention to school personnel and incorporating recurring sessions. Additionally, participants expressed a desire for more time for discussion during the intervention. This feedback aligns with findings from the original SKILLS study with children (Lantz et al. 2021) and from another study on a workshop for teens with ADHD and their parents (Schoenfelder et al. 2020), where both the parents and youth suggested more time for discussion between both peers and families. There is value in keeping SKILLS‐SN short as ADHD is a common condition where the psychoeducational intervention should be offered as a first line of treatment as soon as possible after getting the diagnosis; however, additional sessions could be included for those with extended needs. For these add‐on groups, it is important to consider the appropriate number of participants, as larger groups, as allowed in SKILLS‐SN, might make discussion difficult.

In terms of limited efficacy testing, our hypotheses of preliminary outcomes in a positive direction were not supported. The results from the pre‐ and post‐measurements regarding participants' perceptions of their child's ADHD and their attitudes towards different aspects of treatment showed a positive direction but no significant changes and small effect sizes. Further, no significant change was found in the ratings of ADHD‐related problems. This finding suggests that improvements in ADHD‐related issues take longer than the duration between the two sessions of SKILLS‐SN. As a brief introductory intervention, SKILLS‐SN prioritizes education about ADHD and basic support strategies, rather than aiming to produce immediate changes in attitudes or behaviors. The brevity of SKILLS‐SN is intentional, designed to offer early support shortly after diagnosis with minimal delay or resource demand—an approach that reflects real‐world constraints and promotes accessibility.

### 4.1 Convergence and Divergence Between Qualitative and Quantitative Findings

The combination of qualitative and quantitative data provides a fuller picture of SKILLS‐SN's feasibility and perceived value. While quantitative results showed no significant changes in attitudes towards ADHD or its treatments, qualitative responses suggested psychological and relational benefits such as reduced stress and improved parent–child relationships, not captured by quantitative measures. There was also strong alignment where both data sources highlighted increased knowledge about ADHD as the programme's key benefit. Quantitative ratings showed learning gains, while qualitative comments confirmed these findings with statements like “Provides increased understanding”. These divergences and overlaps underscore the value of integrating qualitative and quantitative methods in feasibility studies.

### 4.2 Limitations and Strengths

To fully evaluate the validity of our findings, some limitations of the current study must be considered. First, the limited number of participants means that a fully representative sample of the targeted group may not be achieved. Additionally, those who chose to participate in the programme were likely interested in gaining more knowledge, while little is known about the characteristics or motivations of those who did not sign up or those who signed up and attended but did not complete the evaluation forms. Based on the collected data, we were unable to calculate the exact proportion of youths whose networks were represented or the exact participation rate. Since the programme was also offered to families who did not participate in the research study, exact group sizes were not consistently recorded. While SKILLS‐SN is designed to be feasible in both small and large group settings, future adaptations—particularly those involving greater interactivity—should consider the potential influence of group size on participant experience. Further, as only a subset of participants provided written feedback, the qualitative findings may reflect the views of those more motivated or engaged. Since the questionnaires were anonymous, the pre‐ and post‐measurements were not paired, limiting the ability to assess individual‐level changes over time. Future studies would benefit from collecting identifiable data, which would enable more robust within‐subject analyses. Additionally, as a feasibility study conducted in only two sites with a self‐selected sample, the findings may not be generalizable to other settings. However, the high retention rate and consistent findings across both regions provide promising indicators for future implementation and support the potential for larger‐scale studies aimed at assessing generalizability. In addition, evaluation forms were sometimes submitted as one per child, even when several participants/relatives attended, which may have compromised the representativeness of the data. Self‐ratings may also be vulnerable to bias. Including more objective measures, such as actual knowledge about ADHD through use of a quiz before and after the intervention, would have increased the validity of our findings. Some qualitative quotes, such as “foundational/basic training,” may allow for both positive and critical interpretations, highlighting the subjectivity inherent in thematic analysis. Lastly, it was not assessed whether the intervention was provided as a hybrid solution or in person for each participant, which is why we cannot draw conclusions regarding potential differences in these experiences. For the future, it would be of value to investigate whether SKILLS‐SN provided online is also feasible and for whom an online version could be more beneficial. Despite these limitations, conducting a feasibility study allows for an early phase of development and fine‐tuning before proceeding to further testing. Having conducted the study at two different sites adds to its strengths by incorporating geographical differences.

## 5 Conclusion

The present study indicates that SKILLS‐SN is feasible, with participants reporting having gained insights regarding ADHD and ways to support their children. The study demonstrates that the intervention can be successfully implemented in clinical settings, as evidenced by participant retention and engagement. However, the impacts of the intervention seem not to lie in symptom reduction or changes in attitudes, although further development and more extensive evaluations are needed to determine the effects of the intervention. In particular, the effect of psychoeducation on treatment adherence has been discussed as an important field for future research (Dahl et al. 2020). Future efforts should aim to improve the programme by incorporating more elements of discussion among the participants. Discussion elements, however, require a smaller group size and could therefore be offered as an optional add‐on for participants interested in more in‐depth discussions. Future efforts should also aim to expand the programme's reach, incorporating feedback for continuous improvement, and exploring the inclusion of school personnel and other significant individuals in the child's life to strengthen the overall support network for children with ADHD. A valuable direction for future research could be to include a control group, such as parents receiving only written materials, to better evaluate the effectiveness of the psychoeducational programme compared to minimal intervention.

## Author Contributions

**Martina Isaksson:** conceptualization, methodology, formal analysis, data curation, writing – original draft, writing – review and editing, visualization. **Daniel Ekenberg:** formal analysis, data curation, writing – review and editing. **Elin Håkonsen Martinsen:** validation, data curation, writing – review and editing. **Måns Lööf:** conceptualization, investigation, writing – review and editing. **Johan Isaksson:** conceptualization, methodology, validation, writing – review and editing, supervision, project administration, funding acquisition.

## Ethics Statement

Ethical approval was obtained by the Swedish Ethical Review Authority (dnr. 2018/271; 2023‐06515‐02).

## Consent

The participants were informed about the study and that participation was voluntary. Consent was obtained by filling out the assessments, and no personal identifiers were collected.

## Conflicts of Interest

The authors declare no conflicts of interest.

## Supporting information


**Appendix S1:** Thematic categories and supporting participant quotes from the qualitative analysis (full overview).

## Data Availability

The data that support the findings of this study are available on request from the corresponding author. The data are not publicly available due to privacy or ethical restrictions.
