# Supplementary material for: Feasibility of the Psychoeducational Programme SKILLS for the Child's Social Network for Patients Newly Diagnosed With ADHD: A Mixed‐Method Design Study
Source: Scand J Psychol. 2025 Oct 21;67(2):321–33. doi: 10.1111/sjop.70034 (PMC12984001; doi:10.1111/sjop.70034)
Supplement: Supplementary file 1 — Appendix S1: Thematic categories and supporting participant quotes from the qualitative analysis (full overview). [file SJOP-67-321-s001.docx]

**Appendix I – Thematic categories and supporting participant quotes from the qualitative analysis (full overview)**

| **Theme** | **Subtheme** | **Quotes** |
| --- | --- | --- |
| **Strengths of the programme** | Appreciation for the programme and its educators | - Inspiring! - Very good - Very appreciated! - Satisfied as a whole. A good education! - Fantastic leaders! - Good educators |
|  | Good as introduction and repetition | - Easy to understand! - Have read a lot about it before, so for me very little was new, but for someone without knowledge it is perfect - Very good as an introduction! - We have a very well-functioning contact with the school that see the problems and suggest adaptations before we do :). Experiences us parents as well informed before Skills but good with a repetition - Good with basic knowledge and what modern research shows - Good basic knowledge, it will be interesting with children's SKILLS in fall 2024 - Good as a foundational/basic training if you don't have much knowledge |
|  | Good structure and content | - Very good length of the education - Good structure, clear, good mix of information/facts/community support/book tips. Thank you :) - It's good that us as parents get to attend together and receive information and tips. - Many tips. A lot of information that you learned and can use. - Good and informative in many ways. Very good tips and advice |

| **Theme** | **Subtheme** | **Quotes** |
| --- | --- | --- |
| **Suggestions for development and improvement** | Physical environment | - Poor sound quality in “Pelarsalen” (one of the hybrid healthcare conference rooms, researchers’ notes) - Better/larger screen needed for the speaker/instructor, better sound. Could have been connected from home. Better with 2 large screens. - It would have been better if it wasn't digital. Digital would have been better if we had been sitting at home. But it would have been best if the speaker had been present. I missed quite a bit when the sound was poor. |
|  | Room for discussion | - Discussion-meetings with relatives, get the possibility to talk with others that have experienced different outbursts/support. - SKILLS is okay for me since I already know a lot about ADHD. What I miss, however, are 'parent groups'—tips and tricks for everyday life. A forum for recognition and community. - More discussions in smaller groups. I believe there is a need to discuss and learn from each other. It can be a bit challenging to just sit and listen. Maybe more opportunities should be provided for talking/discussion. - I wish for more conversation and less lecturing. I suggest 4 sessions with the same content but more discussion around the points raised. |
|  | Extending the content | - Would have wished for more information regarding teenagers (Approximately 15-18 years) - Would need deeper knowledge but will try some of the book recommendations. - My child got its diagnosis very late, feels like the education is directed towards children that have been “noticed” earlier. - Would’ve easily continued on a monthly basis - From an activity-leader perspective I wish more info from other perspectives than just parents - I wish SKILLS was also available for those who have ADHD/ADD/AUTISM, meaning the children. - I felt that it mostly focused on children with both letters. My child, who has only ADD + autism, was not discussed as much. In other words, there was more information about those who have hyperactivity included in their diagnosis. - I would have liked to learn more about characteristics and situations that can be difficult and how to handle them - Would have liked to have more information on how medication affects the different characteristics: activity, concentration, etc. |
|  | Distribution to schools | - Mabey suggest as mandatory for those (working?) in school? - The school-personnel should enroll in this - Wish teachers would take part of SKILLS |

| **Theme** | **Subtheme** | **Quotes** |
| --- | --- | --- |
| **Impact of the programme** | Social and psychological benefits | - The knowledge I have received during Skills has decreased my stress. - I have gotten knowledge that have resulted in a better relationship with my daughter (who has ADHD) - For me, it provided a lot both as a parent and in my professional role, as I work in a school. Somewhere, it was a confirmation that I am doing things right both at home and at work |
|  | Increased knowledge | - Provides increased understanding. - Good for everyone to attend in order to increase knowledge about ADHD and how to approach the child. |
